# Investigating the Pharmacological Mechanisms of SheXiang XinTongNing Against Coronary Heart Disease Based on Network Pharmacology and Experimental Evaluation

**DOI:** 10.3389/fphar.2021.698981

**Published:** 2021-07-14

**Authors:** Li-ying Jia, Gui-yun Cao, Jia Li, Lu Gan, Jin-xin Li, Xin-yi Lan, Zhao-qing Meng, Xin He, Chun-feng Zhang, Chong-Zhi Wang, Chun-Su Yuan

**Affiliations:** ^1^ School of Traditional Chinese Pharmacy, China Pharmaceutical University, Nanjing, China; ^2^ Institute of Traditional Chinese Medicine, Shandong Hongjitang Pharmaceutical Group Co., Ltd. Jinan, Jinan, China; ^3^ School of Biomedical Sciences, Faculty of Medicine, The Chinese University of Hong Kong, Shatin, China; ^4^ Tang Center of Herbal Medicine Research and Department of Anesthesia and Critical Care, University of Chicago, Chicago, IL, United States

**Keywords:** coronary heart disease, network pharmacology, pharmacological mechanisms, experimental evaluation, apoptosis

## Abstract

SheXiang XinTongNing (XTN), which is composed of six traditional Chinese herbs, is a commercially available Chinese patent medicine that has been widely used as the treatment of coronary heart disease (CHD). Its mechanisms against coronary heart disease, however, remain largely unknown. This study aimed to investigate the pharmacological mechanisms of XTN against CHD via network pharmacology and experimental evaluation. In this study, GO enrichment and KEGG pathway enrichment were firstly performed for acquiring the potentially active constituents of XTN, the candidate targets related to coronary heart disease, the drug-components-targets network as well as the protein-protein interaction network and further predicting the mechanisms of XTN against coronary heart disease. Subsequently, a series of *in vitro* experiments, specifically MTT assay, flow cytometry and Real-time quantitative polymerase chain reaction analysis, and a succession of *in vivo* experiments, including Tunel staining and immunohistochemical staining were conducted for further verification. Results showed that Bcl-2, IGF1, CASP3 were the key candidate targets which significantly associated with multiple pathways, namely PI3K-Akt signaling pathway and MAPK signaling pathway. It indicated that the potential mechanism of XTN against CHD may be predominantly associated with cell apoptosis. The *in vitro* experimental results showed that XTN treatment remarkably decreased the apoptotic rate and Bax/Bcl-2 ratio of H9c2 cells. Histological results confirmed that XTN not only effectively alleviated oxidative damage caused by myocardial ischemia but inhibited cell apoptosis. Given the above, through the combined utilization of virtual screening and experimental verification, the findings suggest that XTN makes a significant contribution in protecting the heart from oxidative stress via regulating apoptosis pathways, which lays the foundations and offers an innovative idea for future research.

## Introduction

Cardiovascular disease, as a leading cause of deaths worldwide, has led to 17.3 million deaths globally (Townsend et al., 2016). Most cases of cardiovascular diseases-induced death and disability are attributed to coronary heart disease (CHD), known as one of the most commonly occurred cardiovascular diseases. In developed countries, CHD not only exerted tremendous pressure to the society, but also led to one third of all death cases in the adult population (Roger, 2007; Letnes et al., 2019). Besides, speaking of CHD in developing countries, the mortality of CHD has risen by approximately 10 million from 1990 to 2020 (Sanchis-Gomar et al., 2016). Notably, a new study suggested that patients with cardiovascular disease are more likely to get infected with COVID-19 (Clerkin et al., 2020). Data from the National Health Commission showed that seventeen percent of COVID-19 patients had the comorbidity of CHD (Zheng et al., 2020). High blood pressure, high cholesterol level, diabetes and obesity have been considered as major diagnostic criteria of CHD (Timmis et al., 2018). Women with an onset of type one diabetes before age 10 have a 60 times increased risk of CHD occurrence (Rawshani et al., 2018). Besides, the ApoB level, toxically metal-contaminated environment (Chowdhury et al., 2018) as well as daily life behaviors, such as alcohol intake or cigarette consumption (Hackshaw et al., 2018) can also contribute to a observable growth in the incidence of CHD cases (Do et al., 2013). Apart from those, it has been reported that HIV infection caused a substantially increased risk of CHD (Hsue and Waters, 2019). Percutaneous coronary intervention, coronary artery bypass grafting and medication therapy, specifically nitrates, beta receptor-blockers, stains, as well as antiplatelet drugs have currently been the mainstream therapeutic methods against CHD. Moreover, the abandonment of unhealthy living habit could also alleviate the symptoms of CHD. Regardless of the previously-mentioned treatments against CHD, the therapeutics of CHD still confront with many limitations, more precisely, the lack of comprehensive management, patients’ unwillingness in changing lifestyles and the side effects of medication especially Western medicine. (Mao et al., 2019).

Traditional Chinese medicine (TCM), which is derived from China originally, is extensively characterized by its minor side effects as well as its synergistic therapeutic efficacy. Thus, it has long been widely used in clinical practice and plays indispensable roles in various diseases. In particular, it has broadened the ideas of therapeutic approaches and achieved crucial effects in CHD therapy (Sheng et al., 2014; Tomioka, 2017). According to the TCM theory, the pathogenesis of CHD may include phlegm turbidity, cold coagulation, qi stagnation and blood stasis (Yang et al., 2018). At present, the therapeutic efficacies of many TCM preparations and Chinese patent medicine in relieving chest congestion by activating Qi and removing blood stasis in order to promote blood circulation have shown remarkable effects in CHD. Particularly, SheXiang XinTongNing (XTN) is a commercially available Chinese patent medicine (Reg.No.X0305520) which has been widely used as clinical treatment against CHD for nearly 20 years. XTN is composed of six herbs, including *artificial Moschus* (SX), *Ligusticum chuanxiong* Hort (CX), *Corydalis yanhusuo* W.T.Wang (YHS), *Panax ginseng* C.A.Mey. (RS), *Borneolum Syntheticum* (BP) and *Styrax benzoin* Dryand (SHX). Among them, SX is the principal component that plays a vital role in resuscitation; CX is the dried rhizome of *Ligusticum chuanxiong* Hort. and mainly promotes blood circulation and activates qi (Zhu et al., 2010); YHS (dried tubers of *Corydalis yanhusuo* W.T.Wang) principally regulates qi-flowing, which consequently contributes to pain relief (Luo et al., 2020). As two assistant constituents, CX and YHS combined together play a prominent role in improving levels of the two essentials (qi and blood) in human bodies to eventually alleviate the diseases; BP and SHX are the adjuvant drugs which exert effects in activating brain and clearing away for resuscitation, while they also enhance the effectiveness of other components. The formulae of XTN conform to the theory of TCM and the six kinds of herbs in the formulae have a synergistic effect, so as to achieve a prominent efficacy in relieving CHD symptoms. However, given the characteristics of TCM on possessing multiple components and numerous targets, clarifying the mechanism of TCM has been universally acknowledged to be fairly difficult. Regardless that XTN has been applied in clinical practice for a considerably long period, its fundamental mechanisms against CHD still remain vague.

Network Pharmacology is a systematic approach to integrally investigate mechanisms of drugs and diseases based on the network construction among drugs, constituents, genes, protein targets and diseases (Chen et al., 2018). It not only coincides well with the holistic idea “network target, multicomponent therapeutics” of TCM but also makes a great contribution on predicting potential drugs and screening components, targets as well as pathways of drugs (Yu et al., 2019). In previous study, the network pharmacology has been used in elucidating the key components and mechanisms of TCM involved in their therapeutic effects, such as the mechanisms of Erxian Decoction against TNF-α induced osteoblast apoptosis, the mechanisms of Huayu-Qiangshen-Tongbi formula on rheumatoid arthritis (Wang et al., 2019), the anticancer mechanisms of Compound Kushen Injection against hepatocellular carcinoma (Gao et al., 2018).

Apoptosis, or “programmed death”, is the main form in the early stage of cardiomyocyte death. Suppressing apoptosis is regarded as a promising treatment for CHD. At present, some drugs for CHD have been found to act as apoptosis inhibitors, which is considered as their novel function. For instance, simvastatin is commonly used to regulate lipid metabolism; researches have suggested that it can reduce Bax level as well as Caspase-3 level while up-regulating the level of Bcl-2, thus leading to cardiomyocytes apoptosis suppression, myocardial infarction alleviation and myocardial remodeling (Luo et al., 2014).

In this study, a network pharmacology approach was adopted according to the guidelines published by World Federation of Chinese Medicine Societies (Li, 2021). Then the fundamental mechanisms of XTN against CHD were illuminated through following steps: 1) Screening the chemical components of the six herbs contained in XTN; 2) Predicting the candidate targets of XTN related to CHD; 3) Illustrating a drug-components-targets network and a protein-protein interaction (PPI) network; 4) Functional analysis of XTN for investigating the mechanisms of XTN against CHD. Subsequently, a series of *in vitro* experiments, specifically MTT assay, cell apoptosis assay, JC-1 staining and real-time quantitative polymerase chain reaction (RT-PCR), and a succession of *in vivo* experiments, including Haematoxylin and Eosin (HE) staining, immunohistochemical analysis and TUNEL staining analysis were conducted for further experimental validation. Regarding the findings, our study could provide novel insights into the mechanisms of XTN treating CHD and offer support for further study.

## Materials and Methods

### Active Components Screening

TCMSP (traditional Chinese medicine system pharmacology analysis platform) is a computational platform contains the information of herbs and specially used for systematic pharmacology-based analysis. The identities of the chemical components in XTN were retrieved from TCMSP database (http://tcmspw.com/). The oral availability (OB) and drug-likeness (DL) are considered as two key indicators in drug screen. The OB index represents the percentage of drugs reaching the circulation after oral administration, and the DL index is a qualitative parameter that estimates the similarity between a substance and an existing drug. The OB threshold was set at 40% (OB > 40%) and the threshold of DL was set at 0.18 (DL ≥ 0.18), by which the active ingredients were selected. There are some compounds with strong activity but low OB or DL index in XTN were also incorporate in the study including tetrahydropalmatine, tetrandrine, tetramethylolactone, ligustilide, ligustrazine, ginsenosides (Rb1, Rg1, Rg3, Rd, and Re), muscone, cinnamaldehyde, and Bienyl benzoate. The 3D structures of each active component were retrieved from Pubchem (https://pubchem.ncbi.nlm.nih.gov/).

### Putative and Candidate Targets of Drugs

Furthermore, putative targets hitting every component of XTN were obtained through PharmMapper (http://www.lilab-ecust.cn/pharmmapper/) database. PharmMapper is one of the reverse molecular docking methods developed by reverse pharmacophore mapping based on the characteristics of ligands. The program can quickly obtain drug target information by retrieving four databases, Target Bank, Drug Bank, Binding DB and PDTD. Due to its advantages of fast operation speed and comprehensive target information, it has been extensively used in the research of TCM targets. Specifically, the active components of XTN were uploaded into PharmMapper and underwent reverse pharmacophore matching. The active small molecules were used as probes to search for potential drug targets. When the docking score between molecules and targets, that is, the molecular-target matching degree (Fit Score) is greater than 4.5, it is considered that the target interacts with the chemical components in XTN, thus the putative target is screened out.

The PDB ID of targets was converted into the Gene name with UniProt database (http://www.uniprot.org/).Subsequently, the target genes related to CHD were collected with "coronary heart disease" as key words in genome annotation database platform (Genecards) (https://www.genecards.org/) database, and the common targets were screened as the candidate targets of XTN.

### Network Constructions

To investigate the relationship of XTN and CHD, the drug-components-targets network and protein-protein interaction (PPI) network were constructed with Cytoscape 3.6.0 software (https://cytoscape.org/). In the drugs-components-targets graphical network, the drug, the active molecules and the candidate targets refer to the nodes of network; the edges, which connected the nodes refer to the interactions, and the number of edges in the network refers to the node values.

Further, in order to clarify the interaction between the potential target proteins of XTN, the PPI network of the screened target proteins was constructed on STRING platform (http://string-db.org/). The protein type was set to "Homo sapiens" (human) for operation. The lowest interaction threshold was set to "medium confidence," and other parameters were kept at the default setting. The top 30 core targets were screened and the PPI network was imported into Cytoscape 3.6.0 software for further analysis. The size of the nodes was adjusted in accordance with the connection degrees (the greater the connection degree is, the closer the node is related to other nodes, and the more important it is in the network).

### Pathway Enrichment and Biological Functional Analysis

The annotation, visualization and integrated discovery database (DAVID) database (https://david.ncifcrf.gov/) is a biological information database that integrates biological data and analytical tools together, which can be used for pathway analysis and biological function analysis. The KEGG signal pathway enrichment analysis and GO biological process enrichment of the candidate targets were carried out by DAVID database. The biological processes and pathways with *p* < 0.01 were selected and sorted in line with the number of enriched genes from large to small, then the top 20 biological processes and pathways were chosen for visualization analysis.

## Experimental Verification

### Chemicals and Reagents

SheXiang XinTongNing tablets (Reg.No.X0305520, Lot.No.1905001) were provided by Shandong Hong Ji Tang Pharmaceutical Group Co., Ltd. (Shandong, China). SheXiang BaoXin pills (BXW) (Lot.No.200106) was purchased from Shanghai Hehuang Pharmaceutical Co.,Ltd. (Shanghai, China). Dulbecco's modified Eagle's medium (DMEM) and Fetal bovine serum (FBS) were purchased from Invitrogen Gibco Inc. (Carlsbad, CA, United States). Hydrogen peroxide (H_2_O_2_) was obtained from Nanjing Chemical Reagent Co., Ltd. (Jiangsu, China). MTT [3-(4,5-dimethyl-2-thiazolyl)-2, 5-diphenyl-2-H-tetrazolium bromide] was purchased from Jiangsu Keygen biotechnology Group Co., Ltd. (Jiangsu, China). FITC Annexin V/PI Apoptosis Detection kit and Mitochondria Staining kit were purchased from Lianke Bioscience (Jiangsu, China). RNA isolater Total RNA Extraction Reagent, AceQTM Universal SYBR qPCR Master Mix and HiScript II Q Select RT SuperMix for qPCR (+gDNA wiper) were purchased from Vazyme (Jiangsu, China).

### Cell Culture

Adult rat cardiomyocytes (H9c2 cells) were obtained from the pharmacological research center of China Pharmaceutical University and cultured in DMEM high glucose medium containing 10% FBS antibiotics (100 U/ml penicillin and 0.1 mg/ml streptomycin) and maintained in a 37°C, 5% CO_2_ incubator.

### Cell Viability

According to the results of pre-experiments, XTN had no toxic effects on H9c2 cells at the concentration below 5 mg/ml. Three doses with significant effects were selected: 200, 400, and 800 μg/ml for subsequent experiments. The H9c2 cells were seeded in a 6-well plate (8×10^4^ cells/well) and incubated for 24 h. Then the cells were divided into six groups: control group cultured in serum free complete DMEM; model group cultured in serum free complete DMEM; XTN groups treated with 200, 400, and 800 μg/ml of XTN respectively and BXW group treated with 400 μg/ml of the positive drug BXW. After incubation for 24 h, every groups except control group were treated with 1000 μM of H_2_O_2_ for 1 h. Then, 20 μL of 5 mg/ml MTT solution was added to each well and followed by 4 h incubation. The supernatant was discarded and 150 μL of DMSO was added. In the end, a microplate reader was used to examine cell viability at an absorbance of 492 nm.

### Apoptosis Assay

Cell apoptosis was detected by flow cytometry with Annexin V-FITC/PI apoptosis kit. After treatment of drugs for 24 h and 1000 μM of H2O2 for 1.5 h, 1 × 105 cells in each group were collected and then resuspended in 500 μL binding buffer. Within half an hour prior to detection with flow cytometry (BD FACS Celesta), 5 μL of Annexin V and 10 μL of PI solution were added into the binding buffer in the dark. The apoptotic rates were analyzed with Flowjo 10.4 software.

### Measurement of Mitochondrial Membrane Potential (ΔΨ)

The changes of mitochondrial membrane potential were detected by Mitochondria Staining kit according to the manufacturer’s protocol. The decline of mitochondrial membrane potential is a landmark event in the early stage of apoptosis. JC-1 is an ideal fluorescent probe widely used to detect mitochondrial membrane potential. When the mitochondrial membrane potential is high, JC-1 accumulates in the mitochondria and produces red fluorescence. Otherwise, JC-1 is in the monomer form and can produce green fluorescence. The relative ratio of red and green fluorescence is commonly used to assess the mitochondrial membrane potential. H9c2 cells from different groups were incubated in JC-1 (2 μM) at 37°C with 5% CO_2_ for 30min. After resuspending with PBS, the cells were analyzed by flow cytometry.

### Real-Time Quantitative Polymerase Chain Reaction (RT-qPCR) Analysis

Total RNA from H9c2 cells was extracted with RNA isolater Total RNA Extraction Reagent. Primers for Bax, Bcl-2 and GAPDH were synthesized by Sangon Biotech (Shanghai, China) as follows: Bax: forward: 5′-GTG​GGA​TGG​GCT​TCA​GGA​ACA​AC-3′, reverse: 5′-TCC​AAG​GTC​AGC​TCA​GGT​GTC​TC-3′; Bcl-2: forward: 5′-TTT​CCT​GTT​GCT​GGC​TGG​TTC​TG-3′, reverse: 5′-AGA​CTG​CTG​TAG​TGG​ACA​CCT​GAG-3′; GAPDH: forward: 5′-GAC​ATG​CCG​CCT​GGA​GAA​AC-3′, reverse: 5′-AGC​CCA​GGA​TGC​CCT​TTA​GT-3′. The cDNA was amplified from 1 µg of total RNA using the HiScript II Q Select RT SuperMix forqPCR. Then the gene expression was quantified with AceQTM Universal SYBR qPCR Master Mix under the following reaction conditions: 5 min at 95°C, followed by 40 cycles of 10 s at 95°C, and 30 s at 60°C. GAPDH was used as an internal reference. All data were analyzed through the 2^−∆∆CT^ method.

### Animal Grouping and Establishment of Acute Myocardial Ischemia (AMI) Model

Animals: Healthy Male Sprague-Dawley (SD) rats (250–280 g) were obtained from Qinglong Mountain Animal Breeding Farm (Jiangning District, Nanjing) (Animal permit Number: SCXK (Zhe) 2019-0002, the lab certificate: SYXK (Jiangsu) 2020-0019) and adapted for 1 week before experiment with free access to food and water. All animal procedures were performed in accordance with the animal guidelines and were approved by the Animal Ethical Committee of China Pharmaceutical University.

Model establishment and administration: After anesthetizing the rats with 10% chloral hydrate (0.36 g/kg), fixed the rats and then mechanically ventilated with animal ventilator (Type SA430, SANS, Nanjing, People’s Republic of China). The breathing rate, respiratory ratio and tidal volume of the ventilator were adjusted according to the state of the rats. Then thoracotomy was performed, and the left anterior descending (LAD) was ligated with a 6-0 silk suture. After occluding the LAD coronary artery, closed the chest and sutured the incision. Sham animals were operated similarly without LAD occlusion.

The rats were randomly divided into six groups: sham group, model group, positive control group (SheXiang BaoXinWan, BXW), XTN low dose (0.16 g/kg), XTN middle dose (0.48 g/kg) and XTN high dose (0.96 g/kg). BXW and XTN were administrated via mouth once a day for 4 weeks from the day after ischemic operation. After 4 weeks, all animals were anesthetized. The whole hearts were collected for subsequent morphological examination.

### Haematoxylin and Eosin Staining

The heart tissues obtained from rats were rinsed with cold PBS and then fixed in 4% paraformaldehyde for over 12 h. Then the specimens were cut into four or five sections, each section (4 µm) was enclosed in paraffin and stained with hematoxylin and eosin. The histology changes were observed by upright microscope.

### Tunel Staining

Cell apoptosis of rat cardiomyocytes was measured with a TUNEL Apoptosis Assay Kit (Servicebio, G1507) according to the manufacturer's instructions. Briefly, tissue sections sequentially underwent deparaffinization, rehydration, membrane rupture and incubation of Tunel reaction mixture. Finally, DAB staining was used for the visualization of Tunel reaction.

### Immunohistochemical Analysis

Immunohistochemical staining was carried out for caspase-3 (1:300; Servicebio, GB11009-1) and Bax (1:500; Servicebio, GB11007-1). The tissue sections were deparaffinized and rehydrated, then citric acid (PH6.0) solution was used for antigen retrieval. Endogenous peroxidases were blocked with 3% hydrogen peroxide and nonspecific bindings were blocked with 3% BSA. Next, tissue sections were incubated with primary antibodies at 4°C overnight, followed by incubation of HRP-conjugated second antibodies. In the end, DAB was used for the detection of immunohistochemical reaction. IOD/Area were analyzed using Image-Pro Plus 6.0.

### Statistical Analysis

All data are presented as mean ± standard deviation. Statistical differences among groups were analyzed by one-way ANOVA with the use of GraphPad Prism 7.0. *p* < 0.05 is statistically significant and *p* < 0.01 is considered as statistically high significance.

## Results

### Active Compounds in XTN

Retrieved from TCMSP database, the components of the six herbs in XTN were collected. On the basis of threshold value of OB > 40% and DL ≥ 0.18, 62 active compounds were selected as shown in Table 1.

**TABLE 1:** Active compounds of six herbs and their OB and DL.

Compound	Herb	Chemical	OB	DL
C01	YHS1	Cryptopin	78.74	0.72
C02	YHS2	Dihydrosanguinarine	59.31	0.86
C03	YHS3	(R)-Canadine	55.37	0.77
C04	YHS4	Hyndarin	73.94	0.64
C05	YHS5	Capaurine	62.91	0.69
C06	YHS6	Clarkeanidine	86.65	0.54
C07	YHS7	Corydaline	65.84	0.68
C08	YHS8	Corydalmine	52.5	0.59
C09	YHS9	Corynoline	46.06	0.85
C10	YHS10	Methyl-2-(3,4,6,7-tetramethoxyphenanthren-1-yl)ethanamine	61.15	0.44
C11	YHS11	Dehydrocorybulbine	46.97	0.63
C12	YHS12	Dehydrocorydaline	41.98	0.68
C13	YHS13	Dehydrocorydalmine	43.9	0.59
C14	YHS14	Fumaricine	43.95	0.72
C15	YHS15	Isocorybulbine	40.18	0.66
C16	YHS16	Leonticine	45.79	0.26
C17	YHS17	13-Methylpalmatrubine	40.97	0.63
C18	YHS18	N-methyllaurotetanine	41.62	0.56
C19	YHS19	Pseudoprotopine	53.75	0.83
C20	YHS20	Saulatine	42.74	0.79
C21	YHS21	Stylopine	48.25	0.85
C22	YHS22	Tetrahydroprotopapaverine	57.28	0.33
C23	YHS23	2,3,9,10-Tetramethoxy-13-methyl-5,6-dihydroisoquinolino [2,1-b]isoquinolin-8-one	76.77	0.73
C24	YHS24	Stigmasterol	43.83	0.76
C25	YHS25	Palmatine	64.6	0.65
C26	YHS26	Fumarine	59.26	0.83
C27	YHS27	Bicuculline	69.67	0.88
C28	YHS28	Bulbocapnine	47.54	0.69
C29	YHS29	Quercetin	46.43	0.28
C30	YHS30	Isocorypalmine	35.77	0.59
C31	YHS31	Columbamine	26.94	0.59
C32	CX1	Mandenol	42	0.19
C33	CX2	Myricanone	40.6	0.51
C34	CX3	Perlolyrine	65.95	0.27
C35	CX4	senkyunone	47.66	0.24
C36	CX5	wallichilide	42.31	0.71
C37	CX6	FA	68.96	0.71
C38	CX7	Senkyunolide A	26.56	0.07
C39	CX8	(Z)-Ligustilide	53.72	0.07
C40	CX9	tetramethylpyrazine	20.01	0.03
C41	RS1	Diop	43.59	0.39
C42	RS2	Inermin	65.83	0.54
C43	RS3	kaempferol	41.88	0.24
C44	RS4	Aposiopolamine	66.65	0.22
C45	RS5	Celabenzine	101.88	0.49
C46	RS6	Dianthramine	40.45	0.2
C47	RS7	arachidonate	45.57	0.2
C48	RS8	Frutinone A	65.9	0.34
C49	RS9	Girinimbin	61.22	0.31
C50	RS10	malkangunin	57.71	0.63
C51	RS11	suchilactone	57.52	0.56
C52	RS12	Ginsenoside Rb1	——	——
C53	RS13	Ginsenoside Rg1	——	——
C54	RS14	Ginsenoside Rg3	——	——
C55	RS15	Ginsenoside Rd	——	——
C56	RS16	Ginsenoside Re	——	——
C57	SX1	Muscone	——	——
C58	BP1	asiatic acid	——	——
C59	BP2	bronyl acetate	41.38	0.71
C60	BP3	dipterocarpol	59.3	0.51
C61	SHX1	cinnamaldehyde	41.71	0.76
C62	SHX2	BENZYL BENZOATE	31.99	0.02

### Candidate Targets Screening and Drug-Components-Targets Network

A total of 831 putative targets were screened from PharmMapper. Among them, there were 341 putative targets for YHS, 212 for CX, 182 for RS, 11 for SX, 71 for BP, and 12 for SHX. Further, we obtained 104 candidate targets connected with CHD from Genecards platform. Among them, there are seven for SX, 76 for CX, 55 for YHS, 61 for RS, 41 for BP, and nine for SHX. Some of the chemical components are common to different drugs, so the total number of the targets in the six herbs was more than 104.

**FIGURE 1:**
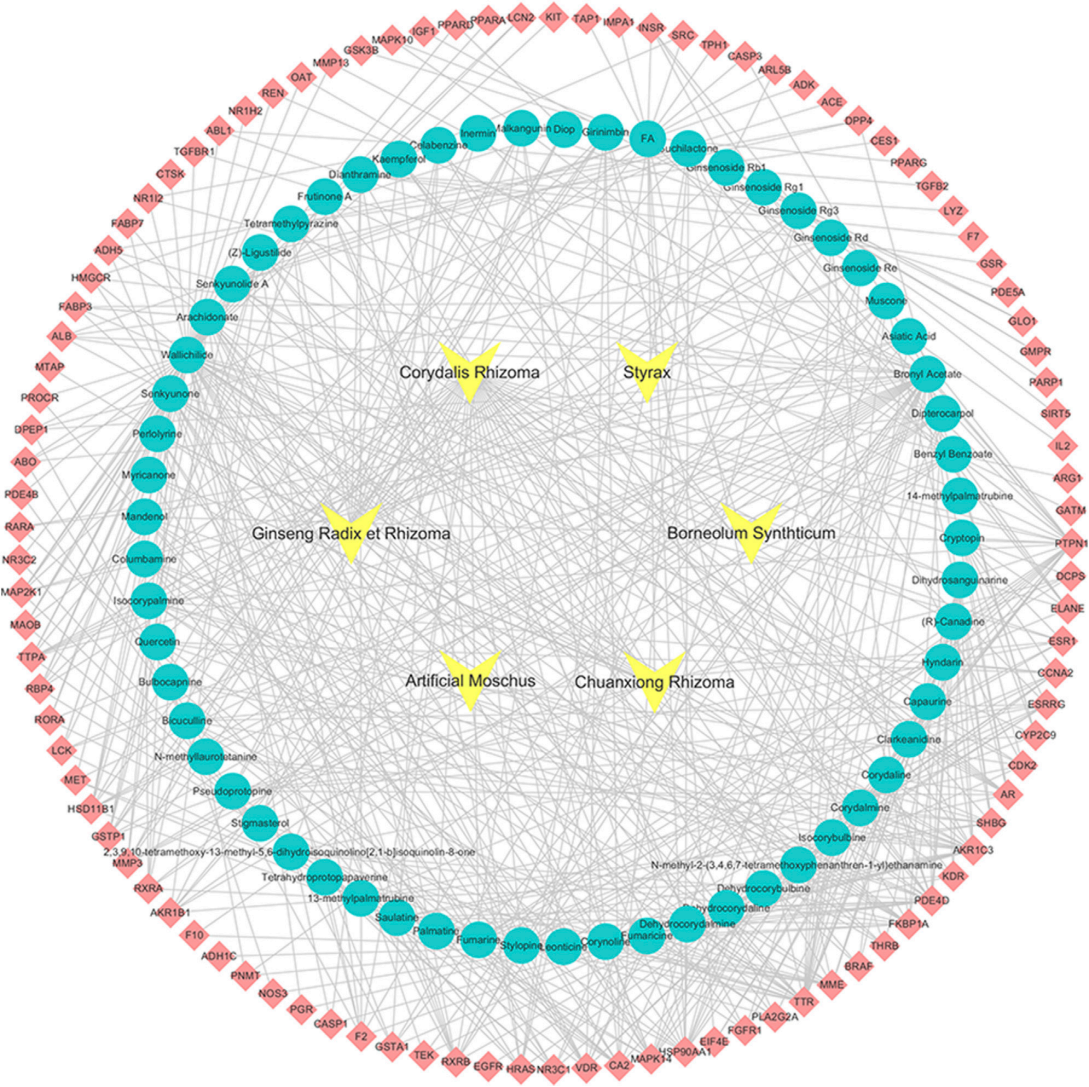
Drug-components-targets network of six herbs in XTN predicted to have 104 candidate targets, the yellow nodes represent the six herbs, the blue nodes represent chemical components and the red are candidate targets.

Additionally, the 62 active compounds and the 104 candidate targets were respectively imported into the Cytoscape 3.6.0 software as the nodes of the network, then the drug-components-targets network was constructed. As shown in Figure 1, the network is comprised of 172 nodes. The nodes in yellow are the six herbs in XTN, the nodes in blue are the chemical components of drugs and the nodes in red are the candidate targets. According to the number of degree and betweenness centrality (BC) of each node, the top four ingredients were Wallichilide, Bronyl Acetate, Senkyunone and Arachidonate, which may be regarded as important components of XTN in the treatment of CHD. In addition, as we can see from the drug-components-targets network, a variety of active ingredients acted on one or more targets, reflecting the multi-component and multi-target characteristics of traditional Chinese medicine.

### Construction of PPI Network

**FIGURE 2:**
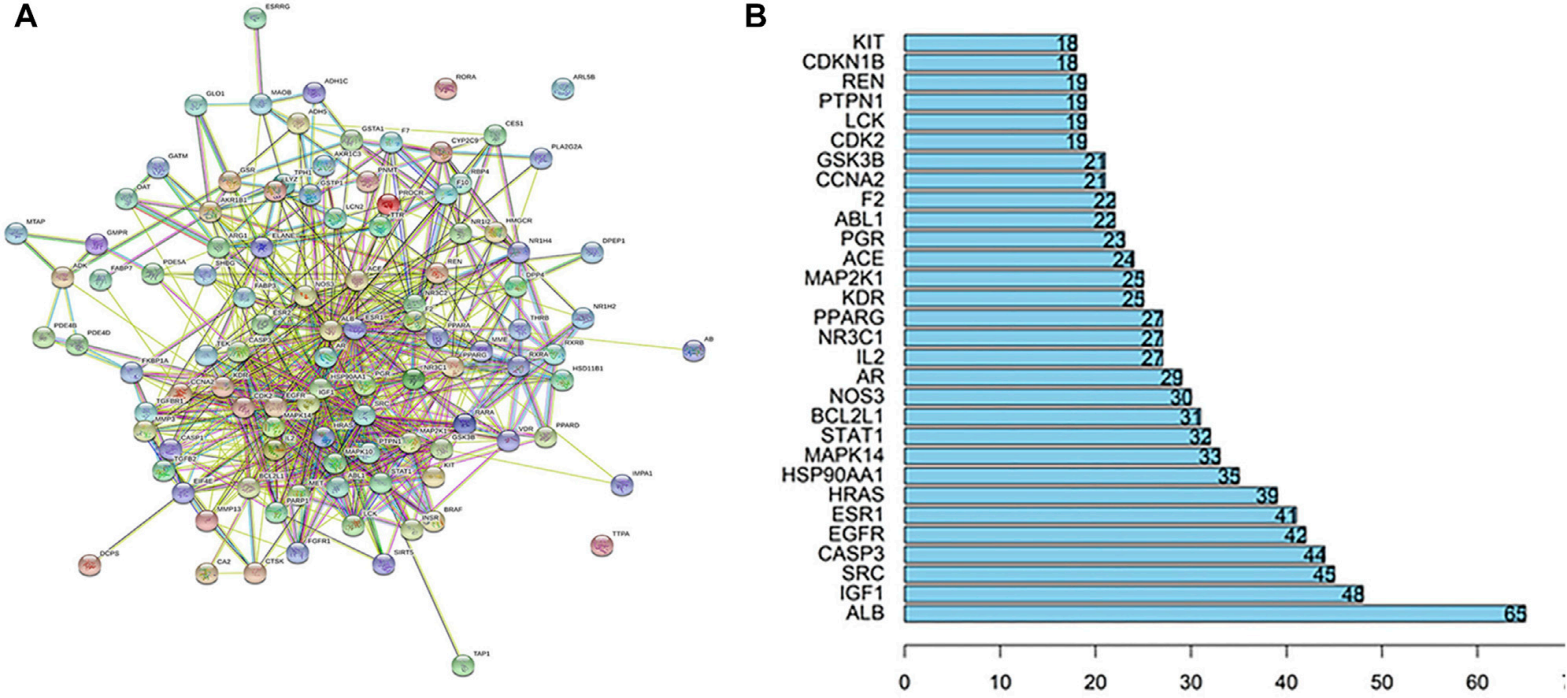
**(A)** PPI network of XTN against CHD. **(B)** The top 30 of core proteins in PPI network.

The STRING database was used to construct PPI network model. The model was then introduced into Cytoscape 3.6.0 software to produce a PPI network diagram (Figure 2A). The top 30 of the core proteins were listed in a bar graph (Figure 2B). Among them, the IGF1, CASP3, EGFR and BCL2l1 are associated with cell apoptosis; the ESR1 and AR are related to hormone regulation; the KDR, ACE and F2 are correlated with blood regulation; the MAPK14, MAP2K1 and GSK3B are related to immunity; the STAT1, NOS3 and IL-2 are associated with inflammation.

### Functional Analysis of Candidate Targets

**FIGURE 3:**
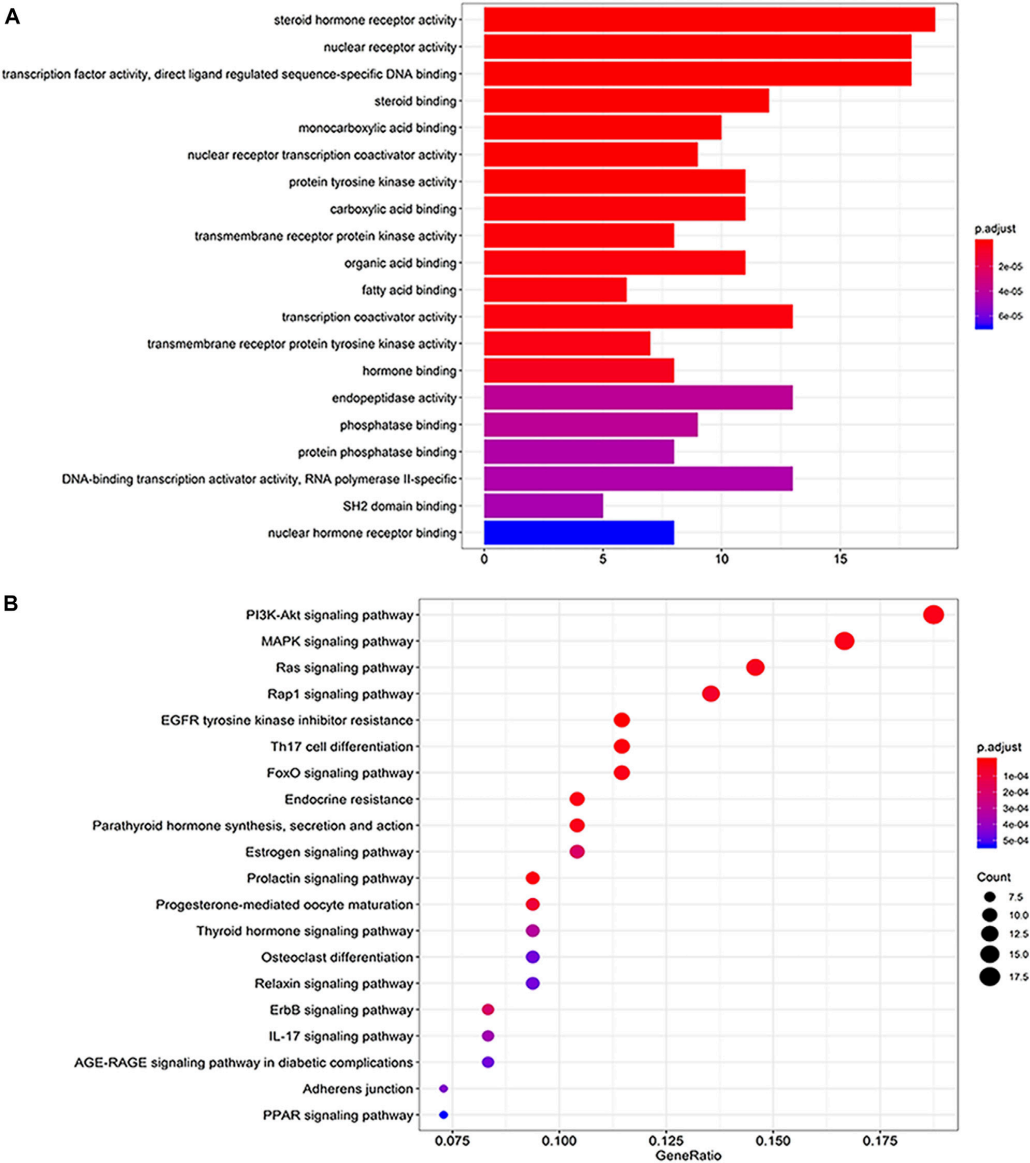
Functional analysis of candidate targets. **(A)** Top 20 of biological process enrichment. Results showed that the candidate targets involved in a variety of biological processes, such as steroid hormone receptor activity, nuclear receptor activity, transcription factor activity, director ligand regulated sequence-specific binding. **(B)** Top 20 of pathway enrichment. Molecular signaling pathways of XTN were highlighted, such as PI3K-AKt signaling pathway, MAPK signaling pathway, Ras signaling pathway, Rap1 signaling pathway.

As exhibited in Figure 3A, the candidate targets of XTN were involved in numerous biological functions. Specifically, there were cellular processes such as nuclear receptor activity and transcription factor activity; immunomodulatory processes such as steroid hormone receptor activity and protein tyrosine kinase activity; emergency response processes including phosphatase binding and protein phosphatase binding; metabolic processes including fatty acid binding and hormone binding.

In the end, the KEGG pathway enrichment shed light on the pathways that the candidate targets may involve in. As shown in Figure 3B, the treatment of XTN against CHD was closely associated with PI3K-AKt signaling pathway, MAPK signaling pathway, Ras signaling pathway, Rap1 signaling pathway, EGFR tyrosine kinase inhibitor resistance, FoxO signaling pathway, Th17 cell differentiation, Endocrine resistance, etc.

### MTT and Flow Cytometry Assay

**FIGURE 4:**
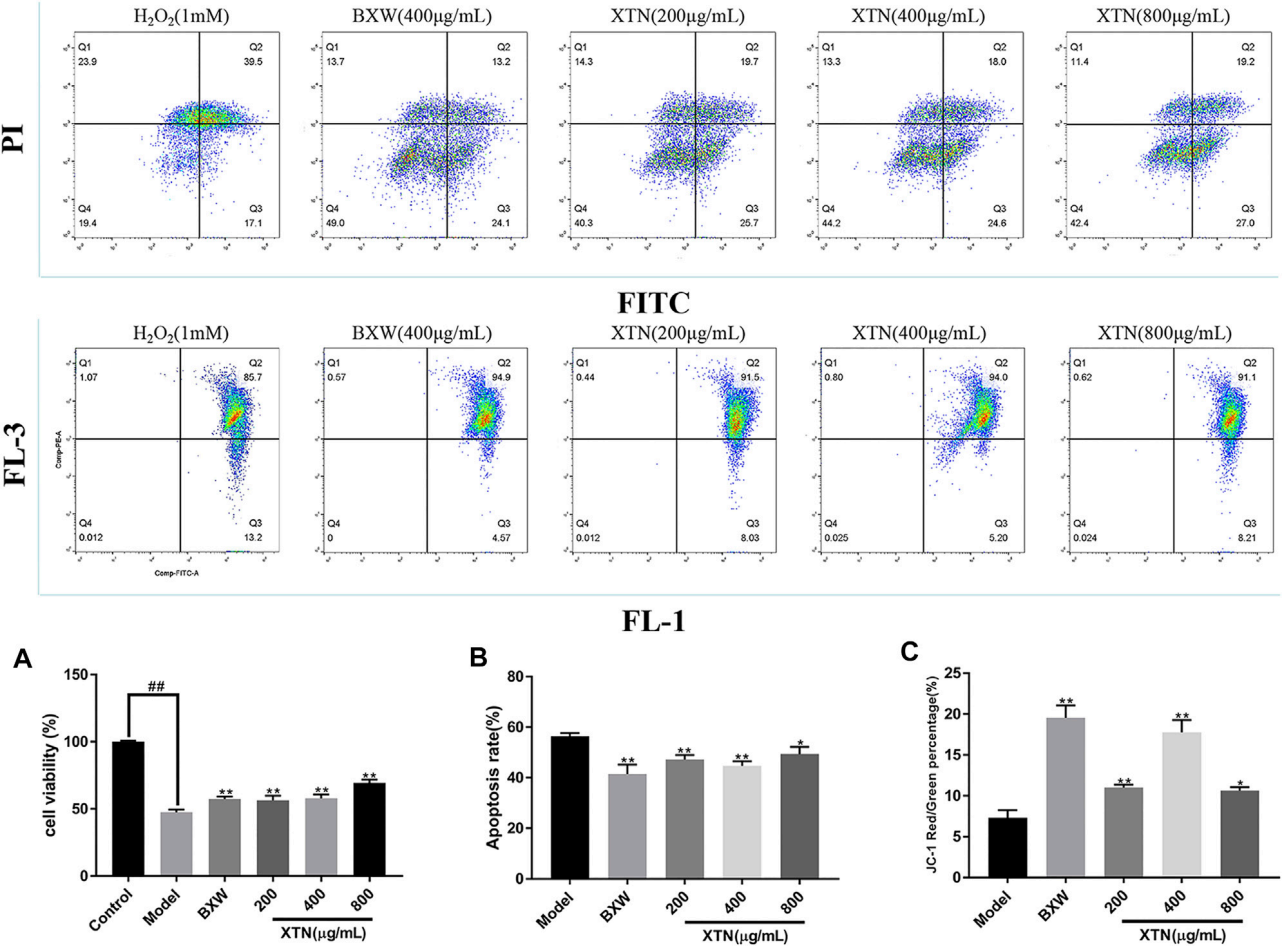
XTN protects H9c2s from H_2_O_2_-induced cardiomyocyte injury. **(A)** Effects of XTN on cell viability **(B)** Effects of XTN on apoptotic rate. **(C)** Effects of XTN on JC-1 red/green percentage (%). Data are expressed as the mean ± standard deviation (*n* = 3). *p < 0.05, **p < 0.01 vs. model group.

In order to validate the effects of XTN on oxidative stress-induced injury in H9c2 cells, MTT assay and flow cytometry were conducted to examine the cell viability and apoptotic rate of H9c2 cells (Figure 4). The cell viability of the model group was significantly lower than that of control group (*p* < 0.01), indicating that the model of H_2_O_2_ induced-oxidative stress injury in H9c2 cells had been successfully established. In comparison with the model group, the cell viability in XTN-treated (200, 400, and 800 µg/ml) groups and positive drug (BXW) group all significantly increased (*p* < 0.01).

Annexin V-FITC/PI staining with flow cytometry was used to investigate the effects of XTN on apoptotic rate of H9c2 cells treated with 1 mM of H_2_O_2_ for 1 h. As results, the model group had a fairly high apoptotic rate, which was up to 57.5%. XTN and BXW slightly inhibited the apoptosis of H9c2 cells. In particular, after being treated with BXW and 400 µg/ml of XTN for 24 h, the apoptotic rates of cells were 40 and 42% respectively. The results showed that XTN may protect cardiomyocytes by suppressing cell apoptosis.

The employment of JC-1 staining with flow cytometry was to detect the alterations of mitochondrial membrane potential (ΔΨ) in H9c2 cells treated with different concentrations of drugs. As shown in Figure 4, compared with the model group, the mitochondrial membrane potential (ΔΨ) in H9c2 cells had slight increase in both XTN-treated and BXW-treated group. Among all the groups with treatments, the XTN (400 µg/ml)-treated group appeared the highest mitochondrial membrane potential (ΔΨ). The mitochondrial membrane potential (ΔΨ) in all groups are as follows: BXW, 94.9%; 200 µg/ml of XTN; 91.5%; 400 µg/ml of XTN, 94.0% and 800 µg/ml of XTN, 91.1%.

### Effects of XTN on mRNA Expression of Bax and Bcl-2 in AMI Rats

**FIGURE 5:**
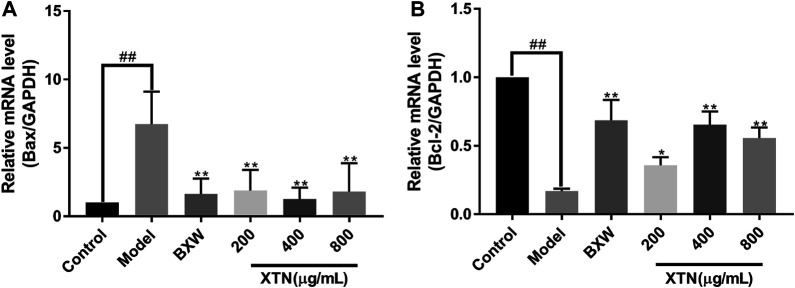
Effects of XTN on mRNA expression of Bax and Bcl-2 in AMI rats. **(A)** Relative mRNA level of Bax. **(B)** Relative mRNA level of Bcl-2. Data are expressed as the mean ± standard deviation (*n* = 6). ^##^ *p* < 0.01 vs. control group; *p < 0.05, **p < 0.01 vs. model group.

To investigate the effects of XTN on cell apoptosis, RT-qPCR analysis was performed to reveal the effect of XTN on Bax and Bcl-2 mRNA expression. Compared with the control group, the expression level of Bax dramatically grew in the H_2_O_2_-treated group, whereas Bcl-2 expression level showed a considerable fall. In contrast, comparing with that of model group, the groups treated with BXW and different concentrations of XTN presented marked decline in expression level of Bax while an obvious up-regulation was observed on Bcl-2 expression level (Figure 5). It suggested that the anti-CHD mechanism of XTN involved apoptosis signaling pathways.

### Effects of XTN on Cardiac Injury in AMI Rats

**FIGURE 6:**
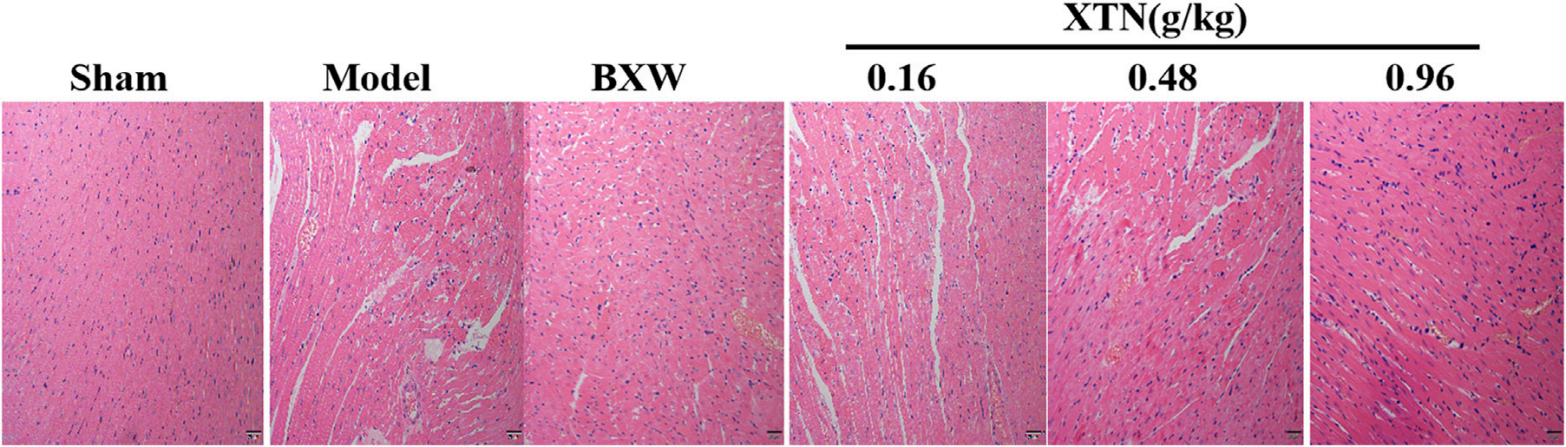
Results of HE staining for cardiac tissues of rats in each group (×200).

HE staining was conducted as a pathological examination to further verify the protective effects of XTN against CHD. As shown in Figure 6, the cardiac tissue of the sham group was complete with clear cell boundaries, regular arrangement, normal cell size and natural morphology, and no infarct lesions was observed; the cardiac tissue of the model group was badly disordered, as indicated by swelling cells, unclear boundaries and enlarged gaps with massive inflammatory cell infiltration, rupture as well as the occurring lysis. In terms of drug treatment, administration of XTN (0.16 mg/kg) slightly ameliorated the myocardial histopathological changes caused by myocardial ischemia. Besides, 0.48 and 0.96 mg/kg of XTN treatment notably relieved the cardiac damage induced by coronary artery ligation with relatively normal cell gaps and distinct boundaries.

### Effects of XTN on Ischemic-Induced Cardiomyocyte Apoptosis

**FIGURE 7:**
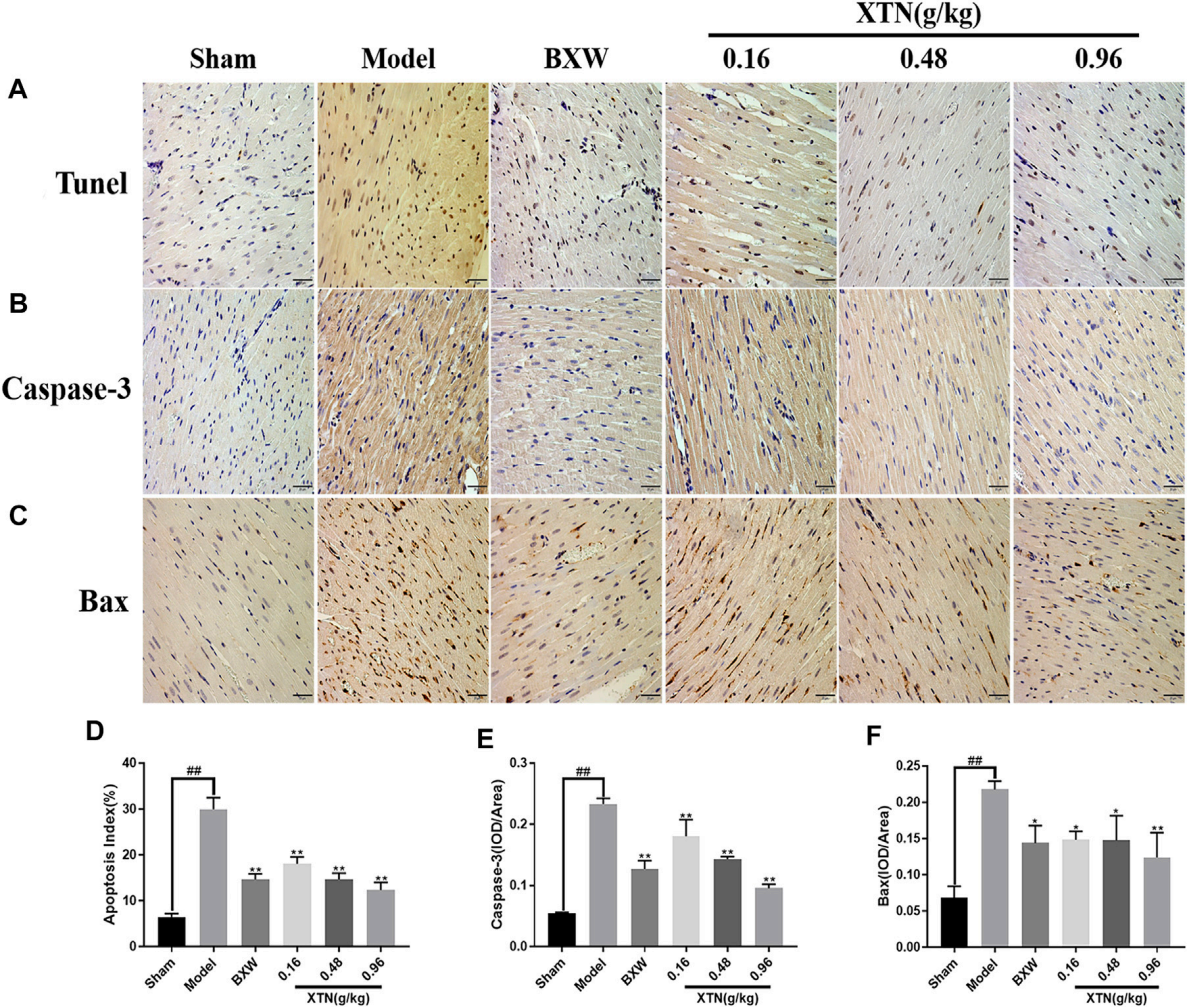
Tunel and Immunohistochemical staining showing the effects of XTN on apoptosis in AMI rats (×400). **(A)** and **(D)** Effect of XTN on apoptosis index. **(B)** and **(E)** Effect of XTN on Caspase-3 protein. **(C)** and **(F)** Effect of XTN on Bax protein. Data represent mean ± SD of three or more independent experiment. ^##^ *p* < 0.01 vs. normal group; *p < 0.05, **p < 0.01 vs. model group.

Tunel staining was employed to assess the protective effects of XTN from cardiomyocyte apoptosis *in vivo*. Immunohistochemical staining was applied to preliminarily explore the mechanisms of XTN protecting rat cardiomyocytes from apoptotic damage. As shown in Figure 7, the apoptosis index of the model group exhibited a considerable increase compared with that of the sham group. Meanwhile, the apoptosis indexes of the groups treated with low-dose, mid-dose and high-dose of XTN all dramatically decreased in comparison with the model group. Besides, the positive protein expression rates of both Caspase-3 and Bax were higher in the model group than that in the sham group (*p* < 0.01). Nevertheless, the positive expression rates of the two apoptotic factors in groups treated with BXW and XTN was lower than that in the model group (*p* < 0.05). In summary, these results suggested that XTN might protect against myocardial ischemic from regulating apoptotic signaling pathways, especially PI3K-Akt pathways inferred from the virtual screening results via network pharmacology methods.

## Discussion

Based upon holistic principles as well as practice of TCM for over 2,000 years, TCM comprehensively focuses on health management that takes all aspects of patients’ life into account rather than just apparent symptoms (Li et al., 2019). “Treatment aiming at its pathogenesis” and “Treating both manifestation and root cause of disease” are the two fundamentals persisted by TCM for more than 2,000 years. The predicament TCM encountered in interpreting its mechanism precisely and being more universally acceptable in clinic application worldwide is owing to the multiple herbs in most of its prescriptions for playing synergistic role all together. (Liu et al., 2018). Network pharmacology corelates the main therapeutic components in prescriptions with the targets of diseases integrally, which may contribute to better elucidate the mechanisms of TCM prescriptions (Yang et al., 2013).

**FIGURE 8:**
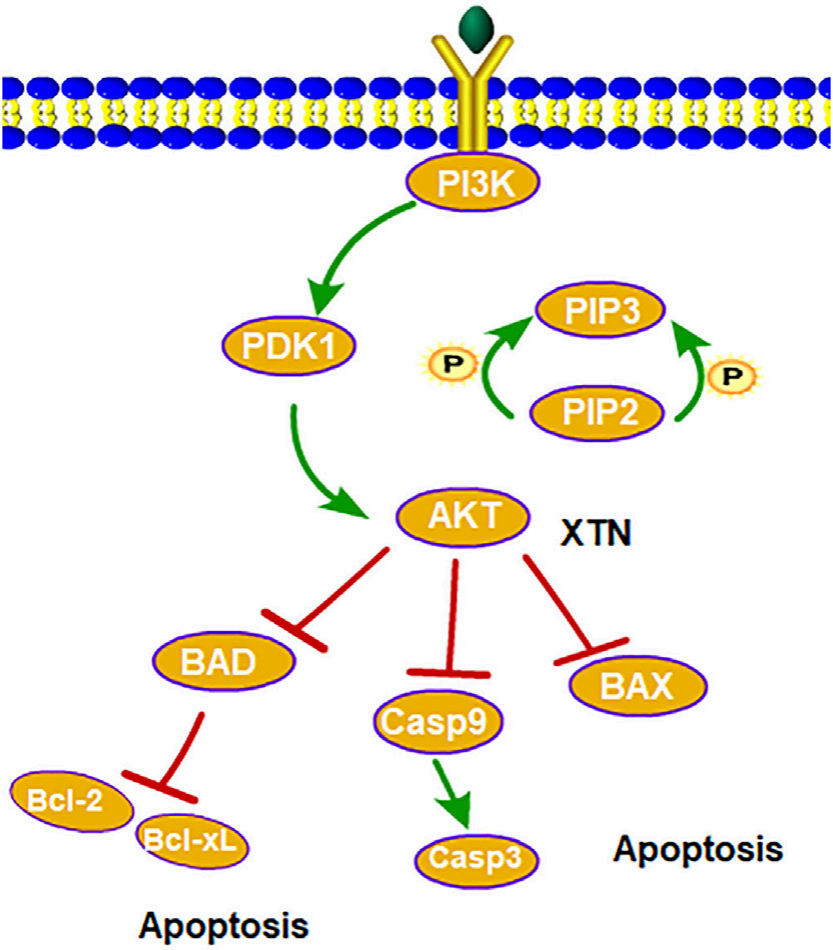
Illustration of the biological process PI3K-Akt pathway caused by candidate targets.

XTN is a classic TCM formula composed of six herbs with remarkable efficacy against CHD. After OB and DL filtering, 62 bioactive compounds were screened out from TCMSP database. The pharmacological activities of these components against CHD have been reported previously. For example, Dehydrocorydalin, an active component of YHS, is known for its anti-platelet, anti-inflammatory and anti-myocardial hypoxia activities (Tan et al., 2019); As a natural alkaloid isolated from YHS, Hyndarin has been proved to exert various effects relating to cardiovascular system, including anti-apoptosis, anti-oxidant activities and improvement of cerebral ischemia-reperfusion injuries (Wu et al., 2007; Han et al., 2012; Mao et al., 2015). In addition, Muscone is the active compound of SX. Several studies have shown that it has anti-cerebral ischemia and anti-myocardial ischemia activities. Accordingly, this indicated that multiple components in XTN function through multiple targets. Moreover, from PPI network we obtained the core proteins of interests, Casp3 and Bcl2. Casp3 is not only closely related to apoptosis but playing a critical role in apoptotic pathways (Ahmadian et al., 2018). One of the earliest changes of apoptosis is characterized by the emergence of a series of protease-Caspases. Activated caspases cleaves massive intracellular enzymes and causes morphological changes of apoptotic cells; the core of this process is exactly the activation of Casp-3 (Jänicke et al., 1998). Bcl2 is an anti-apoptotic protein in the downstream of PI3K-Akt pathway. Interestingly, as the most important pathway in our KEGG pathway enrichment, the PI3K-AKT signaling pathway has been reported to participate in the signal transduction related to various cell activities, namely proliferation, differentiation and apoptosis (Deng et al., 2019). It balances the pro-apoptotic (Bax, Bad, and Bcl-xs) and anti-apoptotic (Bcl-2, Bc1-xl) in Bcl-2 family via activation of both caspases and Bcl-2 family, thereby tightly regulating cell apoptosis as shown in Figure 8. Meanwhile, as the members of Bcl-2 family, Bcl-2 and Bcl-xl are proteins that not only have remarkable anti-oxidant activity but play a vital role in scavenging free radicals and reducing the production of superoxides (Niture and Jaiswal, 2012).

In the present study, MTT and flow cytometry assays showed that XTN effectively increased the cell viability and reduced H_2_O_2_-induced H9c2 cell apoptotic rate. The expression level of Bax showed a growth companied by a decreased expression level of Bcl-2 after H_2_O_2_ stimulation, whereas XTN treatments with different concentrations all showed the opposite results. This indicates that XTN may exert anti-apoptosis effects through upregulating the mRNA expression level of anti-apoptosis factor Bcl-2 and downregulating the mRNA expression level of pro-apoptosis factor Bax. In the next step, *in vivo* experiments were conducted for further validation. As expected, XTN treatment had significantly reduced the apoptotic rate of cardiomyocytes and alleviated the oxidative damage caused by acute myocardial ischemia. Additionally, both the expression levels of pro-apoptotic protein Bax and Caspase-3 exhibited a significant decline in the XTN treatment groups compared with that in model group. Apoptosis is a kind of programmed cell death under the regulation of a variety of genes. In recent years, more researchers have paid attention to the role of apoptosis in the pathogenesis of CHD. Excessive apoptosis always leads to cell death. Considering that ischemia, hypoxia and oxidative stress are three symptoms of CHD, the mechanism of apoptosis is very crucial for the occurrence and development of CHD. Specifically, there are three typical apoptotic signaling pathways: the exogenous apoptosis signal pathway triggered by transmembrane death receptor of TNF receptor family members, the endogenous apoptosis signal pathway mediated by mitochondria and the ER apoptosis pathway regulated by endoplasmic reticulum stress. Over the years, a handful of researchers have conducted studies on the interplay between the PI3K-Akt pathway and apoptosis, which could provide the orientation for our further research. Wang et al. reported that electroacupuncture possessed a neuroprotective effect by inhibiting neuronal apoptosis via the PI3K/AKT/mTOR pathway under the condition of ischemic stroke (Wang et al., 2020). Zhu et al. demonstrated that the up-regulation of Bcl-2 expression in PI3K-Akt pathway could effectively protect the injury caused by ischemia and hypoxia (Zhu et al., 2016).

In general, our study provides a new direction in the treatment of CHD with XTN. The combined utilization of systematic pharmacology and experimental evaluation has broadened our ideas in CHD medication therapy. Our research demonstrated that XTN decreased the expression levels of Bax and caspase-3 but increased Bcl-2 expression level, suggesting XTN has the capacity of anti-apoptosis and the efficacy on anti-CHD. There are, however, still some limitations in the present study. For instance, according to the results of network pharmacology, although we believed that the effect of XTN on CHD is closely related to cell apoptosis, only the potential effects of XTN on classical apoptotic factors Caspase-3 and Bax/Bcl-2 were explored, whereas the possibility of upstream regulatory pathways such as PI3K-Akt pathway were not considered in the current study. In the future, further study such as Western blot analysis and application of inhibitor against PI3K-Akt should be conducted to verify the regulation of XTN on PI3K-Akt signaling pathways.

## Conclusion

In summary, the mechanism of XTN treating CHD was analyzed systematically through the combined utilization of network pharmacology and experimental validation in this study. It offers new insights into exploring the mechanism of XTN against CHD. Based on the results of network pharmacology, being found widely distributable in a variety of pathways, the active components of XTN and their targets corporately play a synergistic role in the protective and therapeutic effects against CHD. Target proteins combining with pathway enrichment analyses indicates that XTN exerts its anti-CHD effect by regulating multiple pathways including PI3K-Akt mediated cell apoptosis pathway, MAPK mediated anti-inflammatory and angiogenesis pathways and so forth. Furthermore, our research confirmed that XTN had significant effects on not only inhibiting cell apoptosis *in vitro* but protecting cardiac tissues from oxidative stressed-mediated myocardial damage *in vivo*. Further study will be performed to verify the mechanisms of XTN inhibiting cell apoptosis. This study presents a fast, economical and comprehensive method for the studies of XTN from a holistic view, which lays a foundation for further study and clinical practice.

## Data Availability

The original contributions presented in the study are included in the article/Supplementary Material, further inquiries can be directed to the corresponding authors.
